# Efficacy and Safety of Combined Extracts of *Cornus officinalis* and *Ribes fasciculatum* for Body Fat Reduction in Overweight Women

**DOI:** 10.3390/jcm9113629

**Published:** 2020-11-11

**Authors:** Eunkuk Park, Chang Gun Lee, Jeonghyun Kim, Jae-Heon Kang, Young Gyu Cho, Seon-Yong Jeong

**Affiliations:** 1 Department of Medical Genetics, Ajou University School of Medicine, Suwon 16499, Korea; jude0815@hotmail.com (E.P.); dangsunsang@naver.com (C.G.L.); danbi37kjh@daum.net (J.K.); 2 Department of Biomedical Sciences, Ajou University Graduate School of Medicine, Suwon 16499, Korea; 3 Department of Family Medicine, Kangbuk Samsung Hospital, Sungkyunkwan University School of Medicine, Seoul 03181, Korea; jaeheon.kang@samsung.com; 4 Department of Family Medicine, Seoul Paik Hospital, Inje University College of Medicine, Seoul 04551, Korea

**Keywords:** overweight, *Cornus officinalis*, *Ribes fasciculatum*, body fat percentage, body fat mass

## Abstract

Obesity is a medical condition that presents excessive fat accumulation with high risk of serious chronic diseases. The aim of this clinical trial is to investigate the anti-obesity effects of *Cornus officinalis* (CO) and *Ribes fasciculatum* (RF) on body fat reduction in Korean overweight women. A total of 147 overweight female participants enrolled in double-blinded clinical trial for 12 weeks and 76 participants completed the clinical study. Participants were treated with four CO and RF mixture (COEC; 400 mg per tablet) or four placebo tablets once a day. Obesity associated parameters (body weight, body mass index (BMI), waist circumference, waist-to-hip ratio, body fat percentage and body fat mass) and safety assessment were analyzed. After 12 weeks of COEC treatment, primary outcomes such as body fat percentage (0.76% vs. 0.01%; *p* = 0.022) and mass (1.1 kg vs. 0.5 kg; *p* = 0.049) were significantly decreased. In addition, the results were statistically significant between the COEC and placebo groups, strongly indicated that COEC had anti-obesity effects on overweight women. Secondary outcomes—including body weight, waist and hip circumference, waist-to-hip ratio, body mass index and computed tomography measurement of visceral fat area, subcutaneous fat area, total abdominal fat area and visceral-to-subcutaneous fat ratio—were reduced in COEC-treated group, but no statistical differences were found between the COEC and placebo groups. The safety assessment did not differ between the two groups. These results suggest that treatment of COEC extract reduces body fat percentage and mass in Korean overweight women, indicating it as a protective functional agent for obesity.

## 1. Introduction

Obesity is a multifactorial disease characterized by excessive body fat accumulation with high risk of several chronic disorders including osteoarthritis, liver and kidney disease, sleep apnea, and depression [1]. Obese people are typically considered over 30 kg/m^2^ of body mass index (BMI), a person’s weight (kilograms) divided by the square of the person’s height [2]. High BMI is generally caused by imbalance of energy intake, decreasing physical activity and environmental factors, leading to development of white adipose tissue [3,4].

Obesity is caused by the enlarged size and the amount of fat cells, leading to fat accumulation with undesirable weight gain [5]. Treatment for obesity is a global issue; the most reliable approaches for managing obesity are long-term lifestyle changes, via reducing food intake and increasing physical exercise intervention [6]. Recently, pharmacological medications have been widely used for the treatment of obesity to reduce body weight with medical comorbidities [7]. The major commercialized drugs for weight loss include liraglutide, methamphetamine hydrochloride, bupropion or naltrexone [8]. Despite the various pharmacotherapies for the prevention of obesity are commercially available, the long-term use of medication has limitations in terms of adverse effects and safety.

Plants have been extensively utilized as alternative herbal medicine for numerous diseases, due to their appropriate for long-term use with less side effects than pharmacological drugs [9,10]. The use of herbal plants has been globally increased as a supplements for the treatment of various diseases, including burning sensation, hypertrophy, cardiovascular disease, cancer, and diabetes mellitus [11,12,13]. In addition, European plant-derived healthy diets are recently recommended such as Mediterranean diet and the Nordic diet [14]. Various studies have demonstrated that herbal plants showed beneficial effects on prevention/treatment of overweight or obesity [15,16]. *Cornus officinalis* (CO) and *Ribes fasciculatum* (RF) are the most popular traditional herbal medicines in Asia. Based on the traditional Chinese pharmacopoeia, CO has been used as an oriental medicine more than 2000 years for the medication of kidney and liver dysfunction, oxidative stress, osteoporosis and obesity [17,18,19]. RF is a widely used as herbal medicine for the treatment of sore throat, cold, antidote, and cough [20]. A current study reported that RF enhanced anti-aging effects by increasing longevity and stress resistance in *Caenorhabditis elegans* [21]. Many studies suggested that combination of natural products presented advantages of synergistic complex effects, compared to single treatments [22,23,24,25]. Current studies demonstrated that treatment of CO and RF mixture (COEC) enhanced anti-menopausal effects by increasing estrogenic activity and decreasing ovariectomy (OVX) induced weight gain and osteoporosis [26]. In addition, mixture of CO and RF extracts showed antiadipogenic effects in pre-adipocyte 3T3-L1 cells and high-fat diet (HFD) induced overweight mice [27]. Although many beneficial effects of the COEC extract have been reported, clinical trials using COEC on obesity have not been studied.

The aim of this study is to investigate the efficacy and safety of combined extracts of CO and RF on body fat reduction via randomized, double-blinded clinical trial in Korean overweight women.

## 2. Experimental Section

### 2.1. Participants

A total of 168 women aged 20–65 years, with BMI of 25–30 kg/m^2^ were recruited from Inje University Seoul Paik Hospital (Seoul, Korea). All the subjects were assessed for eligibility to participate following exclusion criteria; presence of any clinical abnormalities including cerebrovascular diseases (cerebral infarction, cerebral hemorrhage, etc.), cardiovascular diseases (angina pectoris, myocardial infarction, arrhythmia, etc.) within last 6 months; use of certain medications (absorption inhibitors, appetite suppressants, or steroid drugs, etc.) that affect body weight within 1 month; uncontrolled hypertension > 160/100 mmHg; fasting blood glucose over 126 mg/dL or random blood glucose over 200 mg/dL; diabetes patients treated with oral hypoglycemic agents or insulin; increased creatinine, aspartate aminotransferase (AST), or alanine aminotransferase (ALT) levels in blood; severe gastrointestinal disorders; unable to exercise due to myoskeletal disorders; sensitivity to any products containing CO or RF ingredients; pregnancy; subjects who participated or planned to other clinical study within last month. All the participants were provided written informed consent for clinical study. Among them, 21 did not meet the study criteria and were excluded from the study. The 147 remaining participants were given their own Arabic number in order of visit and randomly divided into the COEC group or the placebo group in 1:1 ratio for balanced experimental group using randomization program in SAS^®^ system (Version 9.4, SAS Institute, Cary, NC, USA).

### 2.2. Treatment Material

All the manufacturing process of COEC and placebo tablets were conducted in CosmaxBio Company (Seoul, Korea). Briefly, CO was obtained from Icheon and Yangpyeong (Gyeonggi-do, Korea), and RF was obtained from Goesan (Chungcheongbuk-do, Korea). The CO and RF were air-dried and extracted with ethanol solution. The extracts were filtered for debris removal, and the filtrate was concentrated by evaporator. Finally, the CO and RF extracts were lyophilized and stored at −20 °C before the tablet preparation. COEC tablets were made with the CO and RF extract mixture (400 mg), and the placebo tablet contained the same amount of maltodextrin. For clinical study, the subjects were provided either COEC or placebo tablets to take two tablets for twice a day (1.6 g/day) after meals.

### 2.3. Study Design

Randomized, double-blind, placebo-controlled clinical trial over 12 weeks was conducted between February 2018 and June 2019 with the approval of the Institutional Review Board of Inje University Seoul Paik Hospital (IRB No. PAIK 2017-11-005). Subjects were screened for eligibility to participate in clinical study and scheduled for a baseline visit (0 week). The subjects were randomized and administered COEC or placebo tablets twice a day for 12 weeks. Every four weeks, anthropometric parameters and blood chemistry analysis were assessed, then efficacy evaluation was measured at the end of the study (12 weeks). All subjects and investigators were blinded throughout intervention period. Medication compliance was monitored by examining the remaining tablets at every visit. The subjects in both groups were instructed to reduce energy consumption by 500 kcal/day from their usual diet and to perform physical activity equivalent to 300 kcal/day or more. On the day of each visit, all subjects were asked to submit a diary of their food intake and physical activities for at least 3 days. A trained dietitian inspected all the records and counseled on diet and exercise for the subjects.

### 2.4. Efficacy Evaluation

At the baseline (0 week) and end of the study (12 weeks), total body fat percentage, body fat mass, and lean body mass were determined by dual-energy X-ray absorptiometry (DEXA; Lunar Prodigy Advance, GE Healthcare, IL, USA). Visceral fat area, subcutaneous fat area and total abdominal fat area were evaluated by computed tomography (CT; Light Speed VCT XTE, GE, Japan). Anthropometric parameters such as body weight, hip circumference, waist circumference and BMI were analyzed by the investigator. Blood samples were collected after 12 h of fasting, and lipid panels including total cholesterol, high-density lipoprotein (HDL)-cholesterol, low-density lipoprotein (LDL)-cholesterol, triglyceride and adiponectin) were examined by blood chemistry analysis.

### 2.5. Safety Assessment

For safety assessment, blood electrolytes (Na^+^, K^+^, Cl^−^, and Ca^2+^), and liver function test (AST, ALT, gamma-glutamyl transferase [γ-GTP] alkaline phosphatase [ALP], total protein, albumin, glucose, and total bilirubin), and renal function test (creatine kinase [CK], creatinine, blood urea nitrogen [BUN], and uric acid) were examined between baseline (0 week) and the end of study (12 weeks). All participants were monitored for abnormal events at each visit.

### 2.6. Statistical Analysis

Statistical analyses were performed with the statistical software SAS^®^. The primary outcomes were change in body fat percentage and body fat mass from baseline to 12 weeks, and the secondary outcomes were change in body weight, waist circumference, hip circumference, waist-to-hip ratio, BMI, lean body mass, visceral fat area, subcutaneous fat area, total fat area, visceral-to-subcutaneous fat ratio, total cholesterol, HDL-cholesterol, LDL-cholesterol, triglyceride and adiponectin from baseline to 12 weeks. Efficacy evaluation in each group between 0 week and 12 weeks was assessed by paired *t*-test. The differences between groups at each time point were determined by analysis of covariance (ANCOVA), adjusted by baseline results. All data were presented as mean ± standard deviation (SD), and the * *p* < 0.05 was considered to be statistically significant.

## 3. Results

### 3.1. Inclusion and Exclusion of Participants and Baseline Characteristics

**Figure 1:**
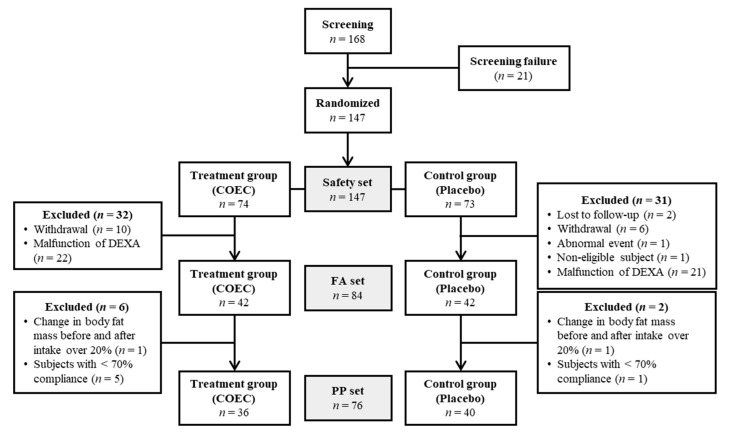
Overall flow diagram of the clinical study. FA, full analysis; PP, per-protocol.

To examine the effects of COEC on obesity in Korean women, a total of 147 overweight female participants was screened in double-blinded clinical trial for 12 weeks. Subjects were randomly divided into the COEC treatment (*n* = 74) and placebo (*n* = 73) groups. Sixty-three participants were excluded because of withdrawal (*n* = 16), lost to follow-up (*n* = 2), abnormal event (*n* = 1) and non-eligibility (*n* = 1), especially forty-three subjects were excluded, because of the malfunction of DEXA equipment (Figure 1). Additional eight subjects were excluded, due to change of body fat mass before and after taking over 20% (*n* = 2) and subjects with less than 70% compliance (*n* = 6) (Figure 1). At the end of the 12-week clinical trial, seventy-six subjects, COEC treatment (*n* = 36) and placebo (*n* = 40) groups, completed the clinical study. No significant differences in baseline demographic characteristics were found between the treatment and control groups. In addition, the number of smokers, alcohol consumption and height did not differ between treatment and control groups (Table 1).

### 3.2. Efficacy Assessment

**Figure 2:**
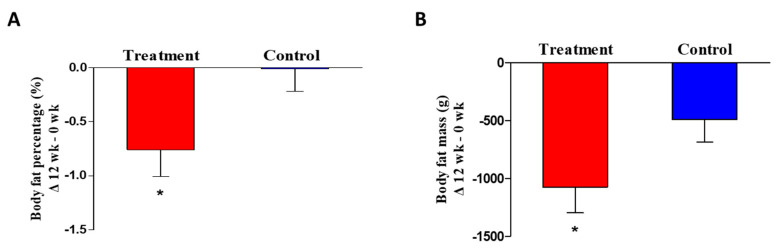
(**A**) body fat percentage, (**B**) body fat mass between treatment (COEC) and control (placebo) groups at 12 weeks. * *p* < 0.05 vs. control (paired *t*-test).

Following 12 weeks of clinical trial, body fat percentage (%), body fat mass (g), and lean body mass (g) were measured by DEXA, and visceral fat area (cm^2^), subcutaneous fat area (cm^2^), total abdominal fat area (cm^2^) and visceral-to-subcutaneous fat ratio were analyzed by CT scanner. As a result, body fat percentage (−0.76 ± 1.48; *p* = 0.004 by paired *t*-test) and mass (−1072.78 ± 1339.04; *p* = 0.001 by paired *t*-test) were significantly reduced in the COEC treatment group (Table 2). Moreover, the changes of body fat percentage (*p* = 0.030 by paired *t*-test, *p* = 0.014 by ANCOVA adjusted baseline) and mass (*p* = 0.049 by paired *t*-test, *p* = 0.037 by ANCOVA adjusted baseline) between two groups were statistically significant after 12 weeks of intervention (Figure 2). In addition, the treatment group showed a significant decrease in lean body mass (−406.83 ± 1067.27; *p* = 0.028 by paired *t*-test), visceral fat area (−12.76 ± 18.51; *p* = 0.001 by paired *t*-test), total abdominal fat area (−19.79 ± 41.14; *p* = 0.006 by paired *t*-test) and visceral/subcutaneous fat ratio (−0.04 ± 0.11; *p* = 0.029 by paired *t*-test). However, the lean body mass, visceral fat area, subcutaneous fat area, total abdominal fat area, and visceral/subcutaneous fat ratio did not differ between the two groups.

We also evaluated obesity parameters including changes from baseline (12 week–0 week) in body weight (kg), waist circumference (cm), hip circumference (cm), waist-to-hip ratio, and BMI (kg/m^2^) between treatment and control group (Table 3). The COEC treatment group showed a significant decrease in body weight (−1.22 ± 2.14 kg; *p* = 0.001 by paired *t*-test), waist circumference (−2.05 ± 2.89 cm; *p* = 0.001 by paired *t*-test), hip circumference (−1.39 ± 1.94 cm; *p* = 0.001 by paired *t*-test), waist-to-hip ratio (−0.01 ± 0.02; *p* = 0.046 by paired *t*-test) and BMI (−0.49 ± 0.83 kg/m^2^; *p* = 0.001 by paired *t*-test). However, there were no significant differences between the two groups. Furthermore, blood chemistry analysis for total cholesterol (mg/dL), HDL-cholesterol (mg/dL), LDL-cholesterol (mg/dL), triglyceride (mg/dL), and adiponectin (μg/mL) did not differ between the treatment and control groups (Table 3).

### 3.3. Safety Assessment

The safety assessment was evaluated by blood biochemistry and adverse events. Blood parameters were analyzed by hematology tests (RBC, Hb, Hct, WBC, platelets, neutrophils, lymphocytes, monocytes, eosinophils, basophils and MCV) and chemistry tests (AST, ALT, total protein, albumin, glucose, total bilirubin, ALP, Na^+^, K^+^, Cl^−^, Ca^2+^, CK, creatinine, BUN, uric acid, and γ-GTP). Hepatotoxicity markers of ALT (−3.42 ± 8.58 IU/L; *p* = 0.002 by paired *t*-test) and γ-GTP (−2.25 ± 5.23 IU/L; *p* = 0.001 by paired *t*-test) were decreased in COEC treatment. However, no statistical differences of blood safety parameters were observed between the treatment and placebo groups (Table 4). Urine examination including SG, pH, leukocytes, nitrite, total protein, glucose, ketone, urobilinogen, bilirubin, and erythrocytes did not differ between two groups. The participants were confirmed for side effects and observable symptoms at each visit. During the intervention period, several abnormal events were found 22 cases of gastrointestinal problems (14 in the COEC group and 8 in the placebo group), 11 cases of infection (6 in the COEC group and 5 in the placebo group), 5 cases of musculoskeletal disorders (2 in the COEC group and 3 in the placebo group), 5 cases of nervous system disorders (4 in the COEC group and 1 in the placebo group), 5 cases of skin and subcutaneous tissue disorders (1 in the COEC group and 4 in the placebo group), 4 cases of procedural complications (1 in the COEC group and 3 in the placebo group), 3 cases of respiratory disorders (2 in the COEC group and 1 in the placebo group), and 7 cases of other disorders (1 in the COEC group and 6 in the placebo group). However, these abnormal events were considered mild and according to clinical doctors’ opinion, neither moderate nor severe adverse events were found (Table 5), resulting in participants that appeared healthy with no adverse effects or pathological signs at the end of the intervention. During the clinical trial, subjects were asked to perform regular exercise at least three times a week. Total food intake and physical exercise between the two groups were not different.

## 4. Discussion

This study investigated anti-obesity effects of the combined extracts of *Cornus officinalis* (CO) and *Ribes fasciculatum* (RF) by DEXA, CT measurement, anthropometric and safety assessment via a randomized, double-blinded clinical trial in Korean overweight or obese women.

A study reported the mechanisms of action of herbal plants on obesity via inhibiting adipogenesis of adipose tissue, suppressing obesity associated hormones, and inflammation [28]. CO extract increases glucose transport activity by regulating GLUT4 expression, and increasing insulin secretion in pancreatic islet [29,30]. RF extract reduces allergic inflammation response via the reduction of nuclear factor-kappa B activation in macrophage [31]. In addition, a study suggested that combined treatment of multiple natural products showed multi-factorial effects on obesity [32].

Combined treatment of CO and RF has become well-known traditional herbal agents during the past decade. Previous studies showed that combined CO and RF extracts had synergistic effects on anti-menopausal syndrome and anti-obesity [26,27]. CO and RF mixture decreased adipocyte differentiation in 3T3-L1 pre-adipocytes, through the downregulation of adipogenic markers such as *Adipoq*, *Fabp4*, *Pparg*, *Srebp1*, and *Plin1* [26,27]. Differentiation of adipocyte is characterized by lipid accumulation [33], CO and RF mixture also inhibited the number of lipid droplets in the cytoplasm of 3T3-L1 cells. Furthermore, CO and RF mixture prevented both obesity murine models including OVX and high-fat diet induced obesity mice, the well-known features of obesity in murine model. Oral administration of CO and RF mixture reduced total body weight, %fat, white adipocyte size and hepatic steatosis, resulting in inhibition of body weight gain in OVX and high-fat diet induced obesity mice [26,27]. These reports support the clinical trial that CO and RF mixture could present anti-obesity effect on human. In this study, COEC presented the anti-obesity effects associated with body fat inhibition in Korean obese women during a 12-week double-blinded clinical trial. Epidemiological studies showed that the prevalence of obesity is higher in women than in men because of their sexual behavior, exercise, and metabolic differences [34,35]. Excluding social factors, the consistently higher incidence of obesity in women suggests that biological factors such as female hormones which play a pivotal role in the development of obesity. For example, estrogen and estrogen receptors modulate glucose and lipid metabolism, and estrogen deficiency leads to increased food intake, and visceral fat accumulation in women [36]. In addition, obese females had additional risk for osteoarthritis, urinary incontinence, and cancer as well as higher incidence for associated diseases than men [37,38,39]. Therefore, this study was limited to Korean women only for the effects of COEC supplement on obesity.

Hip circumference, waist circumference and BMI has been widely used in reliable assessment for obesity [40]. However, anthropometric parameters represent in part of body fat that maybe lead to inadequate examination because of the patient’s metabolic status [41]. In recent years, DEXA and CT have been emerged as novel clinical trial technique for assessment of obesity because of its precise examination for body fat mass, body fat percentage, lean body mass, and fat area [42,43]. However, DEXA and CT limit the amount of radiation and exposure time during the examination period for the patient’s safety [44]. In the present study, COEC supplementation significantly decreased body fat percentage and body fat mass in the Korean subjects. Although there were no statistically significant differences for another measures between the treatment and control groups, COEC efficiently reduced lean body mass, visceral fat area, total abdominal fat area, visceral/subcutaneous fat ratio body weight, waist circumference, hip circumference, waist-to-hip ratio, and BMI. However, body weight, hip circumference, waist circumference and BMI failed to show significant differences between groups. This is probably due to both treatment and placebo subjects were asked to perform regular exercise at least three times a week, resulting in reduction of body weight, hip circumference, waist circumference and BMI. The results support that placebo group also showed significant different body weight, hip circumference, waist circumference and BMI, consequently no differences between groups.

Obesity is associated with high concentrations of total cholesterol, LDL-cholesterol, and triglycerides and low concentrations of HDL-cholesterol in the blood [45]. Blood lipid levels play an important role in the control of modifiable risk factors, including obesity [46], diabetes mellitus [47], and cardiovascular disease [48]. Many studies suggested that weight loss can improve lipid metabolism via increasing levels of HDL cholesterol and decreasing total cholesterol, LDL cholesterol, and triglycerides [49,50]. However, our results showed that treatment of COEC did not affect blood chemistry of total cholesterol, LDL-cholesterol, HDL-cholesterol, and triglycerides. Although some mild adverse events were occurred for the 12 weeks in the clinical trial, the symptoms of adverse event were not considered to be affected in clinical trial, and results showed that there was no significant difference in incidence rate between treatment and control group. In addition, there was no moderate or severe symptoms over the study period and safety assessment in blood chemistry also showed that the results did not differ between the treatment and control placebo groups. However, there was exclusion of many patients due to the malfunction of DEXA was recognized after DEXA calibration was inaccurate. We decided the exclusion of inaccurate calibrated-patients for the successful completion of clinical trials and did not re-test the patients because several inaccurately measured patients were already completed.

## 5. Conclusions

This is the first study to evaluate the efficacy and safety of COEC on inhibiting body weight and body fat in Korean overweight women; a 12 week, double-blinded, randomized, placebo-controlled clinical trial. COEC treatment significantly decreased body fat percentage and body fat mass in Korean overweight women. However, regular exercise at least three times a week reduced body weight, hip circumference, waist circumference and BMI in both groups, resulting in no statistical differences between groups. The malfunction of DEXA was the high drop-out reason. Our results suggest that COEC might be a potential anti-obesity supplement. However, long-term effects of COEC are required for further study.

## Figures and Tables

**Table 1:** Baseline characteristics of subjects participating in this study.

	Treatment Group (*n* = 36)	Control Group (*n* = 40)	Total (*n* = 76)	*p*-Value
**Age**				
Mean ± SD	48.25 ± 9.66	48.60 ± 10.40	48.43 ± 9.99	0.816 *
Min, max	23.00, 61.00	20.00, 64.00	20.00, 64.00	
**Smoking, *n* (%)**				
Non-smokers	34 (94.44)	39 (97.50)	73 (96.05)	0.600 ^‡^
Former smokers	0 (0.00)	0 (0.00)	0 (0.00)	
Current smokers	2 (5.56)	1 (2.50)	3 (3.95)	
**Alcohol consumption, *n* (%)**				
Non-drinker	25 (69.44)	21 (52.50)	46 (60.53)	0.281 ^‡^
Former drinker	0 (0.00)	0 (0.00)	0 (0.00)	
Current drinker	11 (30.56)	19 (47.50)	30 (39.47)	
**Height (cm)**				
Mean ± SD	158.70 ± 4.56	157.57 ± 5.83	158.11 ± 5.26	0.445 *
Min, max	148.30, 168.90	144.90, 170.00	144.90, 170.00	
**Energy intake (Kcal/day)**				
Mean ± SD	1574.48 ± 498.84	1530 ± 406.79		0.353 *
**Energy expenditure (Kcal/day)**				
Mean ± SD	154.62 ± 150.17	141.32 ± 160.70		0.556 *

*: *p*-value by paired *t*-test. ^‡^: *p*-value by Fisher’s exact test.

**Table 2:** Efficacy assessment by dual-energy X-ray absorptiometry (DEXA) and computed tomography (CT) measurement over the 0–12 weeks intervention.

	Treatment Group (*n* = 36)				Control Group (*n* = 40)				
	0 Week	12 Weeks	Change from Baseline (12 Week–0 Week)	*p*-Value **	0 Week	12 Weeks	Change from Baseline (12 Week–0 Week)	*p*-Value **	*p*-Value ^$^
									(12 Week)
**DEXA measurement**									
Body fat percentage (%)	39.21 ± 4.58	38.45 ± 4.33	−0.76 ± 1.48	0.004	39.67 ± 3.66	39.66 ± 3.73	−0.01 ± 1.33	0.971	0.014
Body fat mass (g)	25,608.97 ± 4466.42	24,536.19 ± 4177.00	−1072.78 ± 1339.04	0.001	25,992.23 ± 3845.81	25,501.30 ± 3949.28	-490.93 ± 1206.69	0.014	0.037
Lean body mass (g)	39,469.53 ± 3821.07	39,062.69 ± 3880.21	−406.83 ± 1067.27	0.028	39,429.78 ± 4265.47	38,658.10 ± 4125.28	−771.68 ± 985.55	0.001	0.120
**CT measurement**									
Visceral fat area (cm^2^)	123.72 ± 42.98	110.96 ± 45.79	−12.76 ± 18.51	0.001	119.32 ± 40.04	112.38 ± 36.05	−6.94 ± 15.51	0.007	0.162
Subcutaneous fat area (cm^2^)	230.36 ± 60.73	223.33 ± 50.88	−7.03 ± 32.82	0.207	223.83 ± 56.75	225.40 ± 51.88	1.58 ± 32.54	0.761	0.304
Total abdominal fat area (cm^2^)	354.08 ± 81.86	334.29 ± 70.41	−19.79 ± 41.14	0.006	343.15 ± 77.04	337.78 ± 67.62	−5.37 ± 40.72	0.409	0.162
Visceral- to-subcutaneous fat ratio	0.57 ± 0.25	0.53 ± 0.28	−0.04 ± 0.11	0.029	0.55 ± 0.19	0.52 ± 0.19	−0.03 ± 0.09	0.021	0.817

**: Compared within groups; *p*-value for paired *t*-test. ^$^: Compared between groups; *p*-value for ANCOVA adjusted baseline.

**Table 3:** Anthropometric and blood chemistry analysis of 12-week treatment (COEC) and control (placebo) group.

	Treatment Group (*n* = 36)				Control Group (*n* = 40)				
	0 Week	12 Weeks	Change from Baseline (12 Week–0 Week)	*p*-Value **	0 Week	12 Weeks	Change from Baseline (12 Week–0 Week)	*p*-Value **	*p*-Value ^$^ (12 Week)
**Anthropometric**									
Body weight (kg)	68.09 ± 5.99	66.87 ± 6.13	−1.22 ± 2.14	0.001	68.45 ± 6.54	67.50 ± 6.77	−0.94 ± 1.59	0.001	0.516
Waist circumference (cm)	91.05 ± 5.73	89.00 ± 5.35	−2.05 ± 2.89	0.001	91.68 ± 6.04	89.70 ± 5.60	−1.98 ± 2.22	0.001	0.752
Hip circumference (cm)	100.18 ± 4.54	98.79 ± 4.01	−1.39 ± 1.94	0.001	101.59 ± 4.15	100.45 ± 4.27	−1.14 ± 1.61	0.001	0.281
Waist-to-hip ratio	0.91 ± 0.04	0.90 ± 0.04	−0.01 ± 0.02	0.046	0.90 ± 0.05	0.89 ± 0.05	−0.01 ± 0.02	0.010	0.687
BMI (kg/m^2^)	27.01 ± 1.55	26.52 ± 1.73	−0.49 ± 0.83	0.001	27.51 ± 1.30	27.12 ± 1.45	−0.39 ± 0.64	0.001	0.537
**Blood chemistry**									
Total cholesterol (mg/dL)	205.42 ± 32.69	208.86 ± 30.61	3.44 ± 22.84	0.371	203.48 ± 33.21	212.63 ± 34.91	9.15 ± 23.58	0.018	0.302
HDL-Cholesterol (mg/dL)	56.19 ± 9.80	56.83 ± 11.47	0.64 ± 7.63	0.618	55.83 ± 13.25	55.43 ± 10.47	−0.40 ± 8.32	0.762	0.492
LDL-Cholesterol (mg/dL)	125.50 ± 28.16	131.14 ± 26.64	5.64 ± 19.82	0.096	119.68 ± 30.68	131.35 ± 32.50	11.68 ± 27.37	0.010	0.421
Triglyceride (mg/dL)	118.44 ± 82.06	104.47 ± 51.37	−13.97 ± 50.40	0.105	139.83 ± 101.64	129.08 ± 99.05	−10.75 ± 103.16	0.513	0.357
Adiponectin (μg/mL)	8.10 ± 3.47	8.33 ± 2.90	0.23 ± 2.80	0.619	7.84 ± 3.26	7.89 ± 2.82	0.05 ± 1.61	0.852	0.527

**: Compared within groups; *p*-value for paired *t*-test. ^$^: Compared between groups; *p*-value for ANCOVA adjusted baseline.

**Table 4:** Safety evaluation in blood chemistry between 0–12 weeks intervention.

	Treatment Group (*n* = 36)			Control Group (*n* = 40)			
	0 Week	12 Weeks	Change from Baseline	0 Week	12 Weeks	Change from Baseline	*p*-Value **
			(12 Week–0 Week)			(12 Week–0 Week)	
AST (IU/L)	24.28 ± 6.53	23.19 ± 4.49	−1.44 ± 6.06	24.62 ± 6.83	23.19 ± 4.91	−1.57 ± 5.59	0.880
ALT (IU/L)	20.66 ± 10.46	17.55 ± 6.66	−3.42 ± 8.58	21.82 ± 11.46	19.83 ± 9.49	−2.44 ± 8.32	0.454
Total protein (g/dL)	7.48 ± 0.39	7.46 ± 0.39	−0.01 ± 0.42	7.50 ± 0.39	7.50 ± 0.35	0.01 ± 0.31	0.566
Albumin (g/dL)	4.42 ± 0.22	4.40 ± 0.24	−0.02 ± 0.25	4.46 ± 0.24	4.46 ± 0.24	0.00 ± 0.19	0.678
Glucose (mg/dL)	93.82 ± 7.90	90.77 ± 6.88	−3.00 ± 9.30	94.21 ± 9.63	93.32 ± 11.84	−1.32 ± 12.28	0.548
Total bilirubin (mg/dL)	0.69 ± 0.25	0.69 ± 0.24	−0.01 ± 0.27	0.61 ± 0.23	0.64 ± 0.23	0.03 ± 0.21	0.445
ALP (IU/L)	62.45 ± 16.47	63.53 ± 16.09	1.14 ± 7.16	61.48 ± 16.70	61.87 ± 14.94	0.24 ± 6.90	0.499
Na^+^ (mmol/L)	138.43 ± 2.18	139.03 ± 1.83	0.72 ± 1.94	138.77 ± 1.72	139.10 ± 1.67	0.27 ± 2.14	0.222
K^+^ (mmol/L)	4.22 ± 0.37	4.17 ± 0.43	−0.07 ± 0.52	4.28 ± 0.38	4.28 ± 0.49	0.03 ± 0.53	0.433
Cl^−^ (mmol/L)	103.70 ± 1.80	104.23 ± 2.01	0.53 ± 1.98	103.86 ± 1.67	104.32 ± 1.77	0.41 ± 2.04	0.844
Ca^2+^ (mmol/L)	9.06 ± 0.37	9.15 ± 0.34	0.10 ± 0.45	9.14 ± 0.38	9.23 ± 0.34	0.09 ± 0.36	0.817
CK (mg/dL)	118.68 ± 163.52	100.30 ± 35.13	−25.31 ± 171.08	109.08 ± 56.49	113.68 ± 48.07	6.05 ± 48.72	0.178
Creatinine (mg/dL)	0.65 ± 0.09	0.67 ± 0.09	0.02 ± 0.07	0.66 ± 0.10	0.69 ± 0.10	0.03 ± 0.06	0.435
BUN (mg/dL)	13.09 ± 3.59	13.59 ± 3.78	0.38 ± 3.36	13.40 ± 4.11	13.63 ± 4.95	0.19 ± 4.17	0.853
Uric acid (mg/dL)	4.71 ± 1.06	4.79 ± 1.02	0.06 ± 0.73	4.66 ± 0.91	4.77 ± 0.91	0.11 ± 0.53	0.598
γ-GTP (IU/L)	18.59 ± 9.60	16.30 ± 6.60	−2.25 ± 5.23	21.40 ± 20.71	21.62 ± 21.52	−0.60 ± 8.88	0.255

**: Compared within groups; *p*-value for paired *t*-test.

**Table 5:** Abnormal events between treatment (COEC) and control (placebo) group throughout 0–12 weeks intervention.

	Treatment Group (*n* = 74)		Control Group (*n* = 73)		Total (*n* = 147)		*p*-Value ^‡^
	N	Incidence (%)	N	Incidence (%)	N	Incidence (%)	
Mild	31	100	31	100	62	100	-
Moderate	0	0	0	0	0	0	
Severe	0	0	0	0	0	0	

^‡^: *p*-value for Fisher’s exact test.

## References

[1] Pi-Sunyer X. (2009). The Medical Risks of Obesity. Postgrad. Med..

[2] Hudda M.T., Nightingale C.M., Donin A.S., Owen C.G., Rudnicka A.R., Wells J.C.K., Rutter H., Cook D.G., Whincup P.H. (2018). Patterns of childhood body mass index (BMI), overweight and obesity in South Asian and black participants in the English National child measurement programme: Effect of applying BMI adjustments standardising for ethnic differences in BMI-body fatness associations. Int. J. Obes..

[3] Chiolero A. (2018). Why causality, and not prediction, should guide obesity prevention policy. Lancet Public Health.

[4] Sørensen T.I.A. (2018). From fat cells through an obesity theory. Eur. J. Clin. Nutr..

[5] Longo M., Zatterale F., Naderi J., Parrillo L., Formisano P., Raciti G.A., Beguinot F., Miele C. (2019). Adipose Tissue Dysfunction as Determinant of Obesity-Associated Metabolic Complications. Int. J. Mol. Sci..

[6] Hall K.D., Kahan S. (2018). Maintenance of Lost Weight and Long-Term Management of Obesity. Med. Clin. N. Am..

[7] Kang J.G., Park C.-Y. (2012). Anti-Obesity Drugs: A Review about Their Effects and Safety. Diabetes Metab. J..

[8] Bray G.A. (1993). Use and Abuse of Appetite-Suppressant Drugs in the Treatment of Obesity. Ann. Intern. Med..

[9] Hensrud N.D. (2000). Pharmacotherapy for Obesity. Med. Clin. N. Am..

[10] Kim M.K., Lee W.Y., Kang J.H., Kang J.H., Kim B.T., Kim S.M., Kim E.M., Suh S.H., Shin H.J., Lee K.R. (2014). 2014 clinical practice guidelines for overweight and obesity in Korea. Endocrinol. Metab..

[11] El-Ishaq A., Alshawsh M.A., Chik Z. (2019). Evaluating the oestrogenic activities of aqueous root extract of Asparagus africanus Lam in female Sprague-Dawley rats and its phytochemical screening using Gas Chromatography-Mass Spectrometry (GC/MS). PeerJ.

[12] Teiten M.-H., Gaascht F., Dicato M., Diederich M. (2013). Anticancer bioactivity of compounds from medicinal plants used in European medieval traditions. Biochem. Pharmacol..

[13] Che C.-T., Zhang H. (2019). Plant Natural Products for Human Health. Int. J. Mol. Sci..

[14] Iriti M., Varoni E.M., Vitalini S. (2020). Healthy Diets and Modifiable Risk Factors for Non-Communicable Diseases—The European Perspective. Foods.

[15] Saito M., Yoneshiro T., Matsushita M. (2015). Food Ingredients as Anti-Obesity Agents. Trends Endocrinol. Metab..

[16] Sun N.-N., Wu T.-Y., Chau C.-F. (2016). Natural Dietary and Herbal Products in Anti-Obesity Treatment. Molecules.

[17] Lee N.-H., Seo C.-S., Lee H.-Y., Jung D.-Y., Lee J.-K., Lee J.-A., Song K.Y., Shin H.-K., Lee M.-Y., Seo Y.B. (2011). Hepatoprotective and Antioxidative Activities of Cornus officinalis against Acetaminophen-Induced Hepatotoxicity in Mice. Evid. Based Complement. Altern. Med..

[18] Kim J.Y., Kim Y.-K., Choi M.K., Oh J., Kwak H.B., Kim J.-J. (2012). Effect of Cornus Officinalis on Receptor Activator of Nuclear Factor-kappaB Ligand (RANKL)-induced Osteoclast Differentiation. J. Bone Metab..

[19] Tian W., Zhao J., Lee J.H., Akanda R., Cho J.H., Kim S.-K., Choi Y.-J., Park B.-Y. (2019). Neuroprotective Effects of Cornus officinalis on Stress-Induced Hippocampal Deficits in Rats and H2O2-Induced Neurotoxicity in SH-SY5Y Neuroblastoma Cells. Antioxidants.

[20] Kim J.K., Im J.S., Kim B.S., Cha D.S., Kwon J., Oh C.H., Ma S.Y., Yu J.H., Nam J.I., Jeon H. (2013). Anti-nociceptive Properties of Ribes fasciculatum. Nat. Prod. Sci..

[21] Jeon H., Cha D.S. (2016). Anti-aging properties of Ribes fasciculatum in Caenorhabditis elegans. Chin. J. Nat. Med..

[22] Shah S.A.A., Akhter N., Auckloo B.N., Khan I., Lu Y., Wang K., Wu B., Guo Y. (2017). Structural Diversity, Biological Properties and Applications of Natural Products from Cyanobacteria. A Review. Mar. Drugs.

[23] Mathur S., Hoskins C. (2017). Drug development: Lessons from nature. Biomed. Rep..

[24] Ulrich-Merzenich G., Panek D., Zeitler H., Wagner H., Vetter H. (2009). New perspectives for synergy research with the “omic”-technologies. Phytomedicine.

[25] Rejhova A., Opattová A., Čumová A., Slíva D., Vodička P. (2018). Natural compounds and combination therapy in colorectal cancer treatment. Eur. J. Med. Chem..

[26] Park E., Lim E., Yeo S., Yong Y., Yang J., Jeong S.-Y. (2020). Anti-Menopausal Effects of Cornus officinalis and Ribes fasciculatum Extract In Vitro and In Vivo. Nutrients.

[27] Park E., Lee C.G., Jeong H., Yeo S., Kim J.A., Jeong S.-Y. (2020). Antiadipogenic Effects of Mixtures of Cornus officinalis and Ribes fasciculatum Extracts on 3T3-L1 Preadipocytes and High-Fat Diet-Induced Mice. Molecules.

[28] Fu C., Jiang Y., Guo J., Su Z. (2016). Natural Products with Anti-obesity Effects and Different Mechanisms of Action. J. Agric. Food Chem..

[29] Qian D.S., Zhu Y.F., Zhu Q. (2001). Effect of alcohol extract of Cornus officinalis Sieb. et Zucc on GLUT4 expression in skeletal muscle in type 2 (non-insulin-dependent) diabetic mellitus rats. China J. Chin. Mater. Med..

[30] Chauhan A., Sharma P., Srivastava P., Kumar N., Dudhe R. (2009). Plants Having Potential Antidiabetic Activity: A Review. Der Pharm. Lett..

[31] Jung J.W., Kim S.J., Ahn E.M., Oh S.R., Lee H.J., Jeong J.A., Lee J.Y. (2014). Ribes fasciculatum var. chinense attenuated allergic inflammation in vivo and in vitro. Biomol. Ther..

[32] Yun J.W. (2010). Possible anti-obesity therapeutics from nature—A review. Phytochemistry.

[33] Fu Y., Luo N., Klein R.L., Garvey W.T. (2005). Adiponectin promotes adipocyte differentiation, insulin sensitivity, and lipid accumulation. J. Lipid Res..

[34] Ogden C.L., Carroll M.D., Curtin L.R., McDowell M.A., Tabak C.J., Flegal K.M. (2006). Prevalence of Overweight and Obesity in the United States, 1999–2004. JAMA.

[35] Yang L., Colditz G.A. (2015). Prevalence of Overweight and Obesity in the United States, 2007–2012. JAMA Intern. Med..

[36] Morselli E., Santos R.D.S., Gao S., Ávalos Y., Criollo A., Palmer B.F., Clegg D.J. (2018). Impact of estrogens and estrogen receptor-α in brain lipid metabolism. Am. J. Physiol. Metab..

[37] Kim I.-H., Chun H., Kwon J.-W. (2011). Gender Differences in the Effect of Obesity on Chronic Diseases among the Elderly Koreans. J. Korean Med. Sci..

[38] Foong K.W., Bolton H. (2017). Obesity and ovarian cancer risk: A systematic review. Post Reprod. Health.

[39] Engin A. (2017). Obesity-associated Breast Cancer: Analysis of risk factors. Adv. Exp. Med. Biol..

[40] Kushner R.F. (2012). Clinical Assessment and Management of Adult Obesity. Circulation.

[41] Heymsfield S.B., Cefalu W.T. (2013). Does Body Mass Index Adequately Convey a Patient’s Mortality Risk?. JAMA.

[42] Goss A.M., Gower B., Soleymani T., Stewart M., Pendergrass M., Lockhart M., Krantz O., Dowla S., Bush N., Barry V.G. (2020). Effects of weight loss during a very low carbohydrate diet on specific adipose tissue depots and insulin sensitivity in older adults with obesity: A randomized clinical trial. Nutr. Metab..

[43] Sadeghi A., Mousavi S.M., Mokhtari T., Parohan M., Milajerdi A. (2020). Metformin Therapy Reduces Obesity Indices in Children and Adolescents: A Systematic Review and Meta-Analysis of Randomized Clinical Trials. Child. Obes..

[44] Mattsson S., Vano E. (2012). ICRP perspective on criteria of acceptability for medical radiological equipment. Radiat. Prot. Dosim..

[45] Jiménez J.M., Carbajo M.-A., Lopez M., Castro M.-J., Ruiz-Tovar J., Garcia S., Castro-Alija M.J. (2020). Changes in Lipid Profile, Body Weight Variables and Cardiovascular Risk in Obese Patients Undergoing One-Anastomosis Gastric Bypass. Int. J. Environ. Res. Public Health.

[46] Ravelli A.C.J., Van Der Meulen J.H.P., Osmond C., Barker D.J.P., Bleker O.P. (2000). Infant feeding and adult glucose tolerance, lipid profile, blood pressure, and obesity. Arch. Dis. Child..

[47] New M.I., Roberts T.N., Bierman E.L., Reader G.G. (1963). The Significance of Blood Lipid Alterations in Diabetes Mellitus. Diabetes.

[48] Frantz I.D., Dawson E.A., Ashman P.L., Gatewood L.C., Bartsch G.E., Kuba K., Brewer E.R. (1989). Test of effect of lipid lowering by diet on cardiovascular risk. The Minnesota Coronary Survey. Arter. Off. J. Am. Hear. Assoc. Inc..

[49] Ooi G.J., Doyle L., Tie T., Wentworth J.M., Laurie C., Earnest A., Cowley M.A., Sikaris K.A., Le Roux C.W., Burton P.R. (2017). Weight loss after laparoscopic adjustable gastric band and resolution of the metabolic syndrome and its components. Int. J. Obes..

[50] Cunha F.M., Oliveira J.A.G., Preto J., Saavedra A., Costa M.M., Magalhães D., Lau E., Bettencourt-Silva R., Freitas P., Varela A. (2015). The Effect of Bariatric Surgery Type on Lipid Profile: An Age, Sex, Body Mass Index and Excess Weight Loss Matched Study. Obes. Surg..

